# 

**Published:** 1923-04

**Authors:** 


### PUBLIC HEALTH IN PARLIAMENT.

The following answers have been given for the Minister of Health.

### HOUSING.

Free Houses.—To Sir E. Stockton: Under the present law there are no restrictions of any kind affecting new houses, or old houses newly converted into separate flats or tenements, provided the houses were erected or converted after April, 1919. No question of decontrolling such houses, therefore, arises.

Housing Statistics.—To Mr. Short: Under the State-assisted Housing Scheme 145,771 houses were completed by local authorities and public utility societies up to the 1st November, 1922. Since that date a further 9,263 were completed by 1st February last, and 20,966 were either under construction or had not been commenced. In addition 39,161 houses have been erected by private builders with the aid of the grant under Section 1 of the Housing (Additional Powers) Act, 1919.

Slum Property.—To Mr. Lansbury: Local Authorities already have wide powers under the Housing Acts of acquiring and reconstructing slum properties by means of schemes which, as soon as approved by the Minister, enable the procedure of the Lands Clauses Acts to be applied. These Acts contain special provisions for dealing with cases where owners of property cannot be found. The question whether further legislation is desirable will be considered in connection with the new Housing Bill.

Small Dwellings Acquisition Act, 1899.—To Mr. Wise: My Right Hon. Friend will call the attention of local authorities to the provisions of the Small Dwellings Acquisition Act when he issues the circular in connection with the new housing proposals. Loans have been sanctioned to enable local authorities to make advances under the Act to the extent of £674,151.

Local Authorities (Advances to Builders).—To Mr. Penny (who asked whether, in the Bill relating to the housing of the working classes and the acquisition of small dwellings, it is proposed to insert a provision to enable local authorities to make advances to builders during the course of erection of approved dwellings for persons who desire to own and live in them): Yes, sir.

Subsidy.—To Mr. Trevelyan Thomson: The Grant under Section 1 of the Housing (Additional Powers) Act has been paid in respect of 39,161 houses, the total amount paid being £9,491,927. Information as to the occupation of these houses is not available.

### NATIONAL HEALTH INSURANCE.

Insurance Committees.—To Dr. Watts: There are 128 Insurance Committees in England with an aggregate membership of 3,130. In 1922 the total expenditure on travelling was £2,085, and the payment for subsistence and loss of remunerative time was £1,658. The National Health Insurance Act, 1921, reduced the minimum membership from 40 to 20, and the maximum from 80 to 40, and the revised limits have not yet been in operation sufficiently long to indicate whether any further reduction in the size of these committees is practicable.

Finance.—To Mr. Rhys Davies: The amounts annually expended in England and Wales out of National Health Insurance funds are approximately: (1) Provision of medical benefit (excluding drugs) by insurance practitioners, £6,520,000; (2) provision of drugs and appliances, £1,280,000. The amounts similarly expended in Scotland are: (1) £870,000, and (2) £160,000. Medical benefit is not provided to insured persons in Ireland.

Sickness Benefit.—To Mr. D. G. Somerville: The latest figures available as to the extent of sickness amongst persons insured under the National Health Insurance Acts indicate that the aggregate amount of sickness during the past year fell short of the expectation on which the Acts were based, and also of the actual experience of any pre-war year. So many factors affect the total expenditure on sickness benefit that it is impossible to assign variations in that figure to particular causes, but there is no direct evidence of an increase of sickness owing to the cause [lack of sufficient nourishment] referred to by the Hon. Member.

Medical Benefit.—To Sir Kingsley Wood (who asked whether, in view of the expiration of the agreements at the end of this year with doctors on the panel lists, steps are being taken to review the present scheme of medical benefit with a view to rendering it more efficient and economical): Yes, sir. The existing arrangements for the medical treatment of insured persons are now being examined in detail by the Insurance Consultative Council of the Minister of Health.

Tuberculosis (Treatment).—To Mr. Rhys Davies: The number of persons recommended for and awaiting treatment for tuberculosis in residential institutions from local authorities in England and Wales on February 1, 1923, was 2,902, on which date 16,393 persons were receiving such treatment, and there were a number of vacant beds. My Rt. Hon. Friend is not aware of the number of notified cases which have not been admitted to residential institutions; but the hon. member no doubt appreciates that not all notified cases of tuberculosis require, or are willing to receive, such treatment.

Institutions.—To Mr. Briant: The institutions approved for the treatment of tuberculosis in England, apart from Poor Law Institutions, contain 19,240 beds. Of these 3,310 beds are in children's institutions, but the remaining 15,930 beds are available for insured persons although, since the termination of sanatorium benefit, no beds are specially reserved for such persons.

Spahlinger Treatment.—To Captain Wedgwood Benn: (who asked the Minister of Pensions whether his attention has been called to the Spahlinger treatment for tuberculosis; and whether it is intended to make this available for ex-Service patients): My Rt. Hon. Friend is informed that no supplies of M. Spahlinger's remedies are yet available in this country. When they become available it will be open to the medical officers responsible for the treatment of ex-Service men to utilise these remedies in the treatment of their patients if they consider it desirable.

Blind Persons (Training Centres, &c.).—To Mr. Graham White: Since the Blind Persons Act, 1920, came into operation four new workshops have been opened and considerable extensions have been made to six existing workshops. One new hostel has been opened and one extended. Training centres are under the control of the Board of Education, but my Rt. Hon. Friend is informed by that department that they have recognised eight new centres and four new hostels for training purposes.

Diabetes (Insulin).—To Dr. Chapple: It is not the case that the poor only are deprived of the advantages of Insulin, which cannot be made available until a supply of known efficiency, potency and safety is attained upon a manufacturing scale. Considerable progress has been made by the Medical Research Council in this direction; and the Council believe that subject to limitation of raw material in particular districts, the successful manufacture of Insulin in the near future is assured. No special grant-in-aid would accelerate production at this stage.

### HOSPITALS.

Voluntary Hospitals (Grant).—To Mr. Gilbert: The whole of the £500,000 grant has not yet been distributed. The Commission allocated £225,000 to hospitals in London, and the whole of that sum has now been disbursed. £275,000 was allocated to the hospitals outside London. Of this sum £181,318 has been paid to date, and the Commission intend shortly to consider the distribution of the balance after the claims in respect of the 1921 deficits have been met. The Commission have not yet made any recommendation as to the continuance of State assistance after the present grant is exhausted.

### WATER SUPPLY.

A Survey of Resources.—To Sir Charles Cayzer: A survey of the water resources and requirements of the country is at present being undertaken with a view to the adoption of a general policy. It would not, however, be practicable to hold up all applications in the meantime so far as there is serious need in any particular areas for additional water supplies.

### RECENT APPOINTMENTS.

### Medical Officers, Chaplains, etc.

Chavasse, Francis Bernard, M.R.C.S., L.R.C.P., M.C.; Honorary Assistant Surgeon to the Eye Department of the Liverpool Eye and Ear Infirmary.

Farrar, Bellwood, M.B., Ch.B.; Tuberculosis Officer Arbroath.

Gimblett, Charles Leonard, M.D., F.R.C.S., senior Clinical Assistant, Westminster Hospital; Part Time Ophthalmic Surgeon, East Ham Education Committee (Caius College, Cambridge).

Mason, John Harold, M.B., Ch.B., M.R.C.S., L.R.C.P., D.P.H., School M.O., Northamptonshire County Council; Assistant School M.O. and Assistant M.O.H. Borough of Northampton.

Murdoch, Georgina, Ashcroft, Coatbridge, Scotland; assistant school M.O., Wigan Education Authority, £500.

Rolleston, Sir Humphry Davy, K.C.B., F.R.C.P.; Chairman of the Central Joint Voluntary Aid Detachment Council.

Shaw, Surgeon Commander Thomas B., M.B., Specialist in Bacteriology, Royal Naval Hospital, Plymouth; Professor of Hygiene and Director of Medical Studies, Naval Medical School, Royal Naval College, Greenwich.

Sophian, George John, M.R.C.S., L.R.C.P.; M.O. for Maternity and Child Welfare Work, Greenwich.

### Public Health and Hospital Appointments.

Allan, G. Grant, M.B., Ch.B., D.P.H. (Edinburgh); Assistant Bacteriologist, Sheffield University.

Eldred, Ernest F., District Sanitary Inspector Acton; Sanitary Inspector, Bury St. Edmund's.

Elkington, H., Sanitary Inspector, Sowerby Bridge.

Gillam, Marion, Nurse under Buckinghamshire County Council; Health Visitor, Taunton (Bradford Royal Infirmary).

Hall, Mary A.; Health Visitor, Manchester City (Bury Union Hospital).

Jones, Margaret; Health Visitor, Manchester City (Crumpsall Infirmary).

Lambert, Sarah J., R.R.C. (Queen Victoria Jubilee Nurses' Institute); County Superintendent, Isle of Wight.

Lewis, H. P., B.A. (Cambridge), B.Sc. (Wales); Assistant Lecturer in Mining Geology, Sheffield University.

Malter, R. H., Sanitary Inspector, Nuneaton.

Milne, Alice; Health Visitor, Manchester City (Crumpsall Infirmary).

North, J. W., Sanitary Inspector, North Manchester.

Owen, C. E., Montgomery County Office; School Nurse, Merioneth Education Committee (London Hospital).

Raimes, C., Chief Assistant Sanitary Inspector, Newcastle-on-Tyne; Chief Health Inspector, Newcastle-on-Tyne.

Tingle, Clara D., M.B., Ch.B. (Sheffield); Demonstrator in Pathology, Sheffield University.

Wilson, A. A., Sanitary Inspector, Dundee.

Woods, G. R., Sanitary Inspector, Norwich.

